# Alone Yet Not Alone: *Frankia* Lives Under the Same Roof With Other Bacteria in Actinorhizal Nodules

**DOI:** 10.3389/fmicb.2021.749760

**Published:** 2021-12-02

**Authors:** Faten Ghodhbane-Gtari, Timothy D’Angelo, Abdellatif Gueddou, Sabrine Ghazouani, Maher Gtari, Louis S. Tisa

**Affiliations:** ^1^Laboratoire Microorganismes et Biomolécules Actives, Faculté des Sciences de Tunis, Université de Tunis El Manar, Tunis, Tunisia; ^2^Institut Supérieur de Biotechnologie de Sidi Thabet, Université de la Manouba, Sidi Thabet, Tunisia; ^3^Unité de Bactériologie Moléculaire et Génomique, Centre Urbain Nord, Institut National des Sciences Appliquées et de Technologie, Université de Carthage, Tunis, Tunisia; ^4^Department of Molecular, Cellular, and Biomedical Sciences, University of New Hampshire, Durham, NH, United States

**Keywords:** actinorhizal symbiosis, microbiome, endophyte, symbiont, plant-growth-promoting bacteria

## Abstract

Actinorhizal plants host mutualistic symbionts of the nitrogen-fixing actinobacterial genus *Frankia* within nodule structures formed on their roots. Several plant-growth-promoting bacteria have also been isolated from actinorhizal root nodules, but little is known about them. We were interested investigating the *in planta* microbial community composition of actinorhizal root nodules using culture-independent techniques. To address this knowledge gap, 16S rRNA gene amplicon and shotgun metagenomic sequencing was performed on DNA from the nodules of *Casuarina glauca.* DNA was extracted from *C. glauca* nodules collected in three different sampling sites in Tunisia, along a gradient of aridity ranging from humid to arid. Sequencing libraries were prepared using Illumina NextEra technology and the Illumina HiSeq 2500 platform. Genome bins extracted from the metagenome were taxonomically and functionally profiled. Community structure based off preliminary 16S rRNA gene amplicon data was analyzed *via* the QIIME pipeline. Reconstructed genomes were comprised of members of *Frankia*, *Micromonospora*, *Bacillus*, *Paenibacillus*, *Phyllobacterium*, and *Afipia*. *Frankia* dominated the nodule community at the humid sampling site, while the absolute and relative prevalence of *Frankia* decreased at the semi-arid and arid sampling locations. Actinorhizal plants harbor similar non-*Frankia* plant-growth-promoting-bacteria as legumes and other plants. The data suggests that the prevalence of *Frankia* in the nodule community is influenced by environmental factors, with being less abundant under more arid environments.

## Introduction

*Casuarinaceae* family comprises four plant genera (*Allocasuarina*, *Casuarina*, *Ceuthostoma*, and *Gymnostoma*) and approximately 86 species and 13 subspecies indigenous to Australia (Steane et al., 2003; Gtari and Dawson, 2011; Diagne et al., 2013; Normand et al., 2014) that are frequently introduced into approximately 150 tropical and subtropical countries where they play an important role for land reclamation, crop protection and as windbreaks (Diagne et al., 2013; Potgieter et al., 2014). Large-scale planting of casuarinas has proven to have a strong impact especially in China, Senegal, Egypt, and Tunisia (Gtari and Dawson, 2011). In arid and semi-arid areas, salinization of soils and groundwater is a serious problem causing a drastic reduction in agricultural production (Munns, 2005; Rengasamy, 2006)^1^. Over 800 million hectares of land throughout the world are salt affected. A common method for dealing with salt stress problems is to reclaim saline soils with multipurpose, fast-growing salt-tolerant tree species such as *Casuarina* tree. Casuarina are notable for high salt tolerance (Tani and Sasakawa, 2003) and have been used as a green barrier. Despite the applied interests, substantial global invasion consequences for *Casuarina* has noted in Florida and the Mascarene Islands (Potgieter et al., 2014).

Actinorhizal plants, like *Casuarina*, form a nitrogen-fixing symbiosis with the Actinobacteria *Frankia* that results in the formation of root nodule structures where the bacteria are located (Chaia et al., 2010; Normand et al., 2014). Besides *Frankia*, actinorhizal plants are known to associate with mycorrhizae including both ectomycorrhizal or endomycorrhizal fungi (Zhong et al., 1995; Duponnois et al., 2003; Diagne et al., 2013; Zhou et al., 2017). The symbioses with *Frankia* and mycorrhizae allow actinorhizal host plants to colonize harsh environmental terrains including highly contaminated, dry, poorly drained, nutrient-poor, and salinized soils (Diagne et al., 2013, 2015).

Besides *Frankia*, several other non-*Frankia* bacteria have been isolated as a by-product of *Frankia* isolation attempts (Ghodhbane-Gtari and Tisa, 2014). Large numbers of other bacteria were collected from these actinorhizal nodules, occupying the same microniche as *Frankia*. Most of these isolates were ignored or discarded as being irrelevant to the plants. However, these non-*Frankia* Actinobacteria have consistently been isolated from several actinorhizal plants including *Casuarina* (Guillén et al., 1993; Niner et al., 1996; Valdes et al., 2005; Ghodhbane-Gtari et al., 2010), and consist of Actinobacteria from *Micromonospora* (Valdes et al., 2005; Ghodhbane-Gtari et al., 2010), *Nocardia* (Ghodhbane-Gtari et al., 2010, 2019) and *Streptomyces* genera (Ghodhbane-Gtari et al., 2010). Similarly, non-symbiotic bacteria, such as *Azospirillum*, *Bacillus*, *Pseudomonas*, and *Streptomyces*, are known to be auxiliary bacteria in several *Rhizobium*-legume symbioses (Iruthayathas et al., 1983; Grimes and Mount, 1984; Li and Alexander, 1990). In this case, both wild and cultivated legume nodules are not exclusively inhabited by rhizobia, but contain diverse assemblages of non-rhizobial bacteria (Dudeja et al., 2012; Aserse et al., 2013; Martínez-Hidalgo and Hirsch, 2017). These nodule non-rhizobial communities may be influenced more by soil type rather plant genotype (Leite et al., 2017).

During the last decade, culture-dependent and –independent approaches have been used to intensively investigate these effects. For legume nodules, rhizobial, and non-rhizobial endophytes communities have been initiated (Martínez-Hidalgo and Hirsch, 2017; Zhang et al., 2018; Mayhood and Mirza, 2021), but very little is known about microbial communities and their structure associated with actinorhizal nodules. Endophytic bacteria can be found between cells, while others are associated in an intimate relation with the plant. For that intimate association to occur, complex relationships are required during the colonization of plant tissues. Plant endophytes are described as having the ability to restrain pathogen infection, to accelerate seedling emergence, and to promote plant growth. Thus, they can be considered as biocontrol agents (Ryan et al., 2008; Eljounaidi et al., 2016; Santoyo et al., 2016).

In this study, we investigated the structure and function of bacterial communities found in *Casuarina glauca* nodules and associated rhizosphere soils and bulk soils from three different bioclimates in Tunisia. We used both high-throughput amplicon (targeting of the 16S rRNA gene) and whole metagenome shotgun sequencing of *C. glauca* nodules found from the different bioclimates: humid, semi-arid and arid condition. For comparative purposes, high-throughput amplicon sequencing was performed on rhizosphere soils, and bulk soils for these three sites.

## Materials and Methods

### Sampling

**FIGURE 1:**
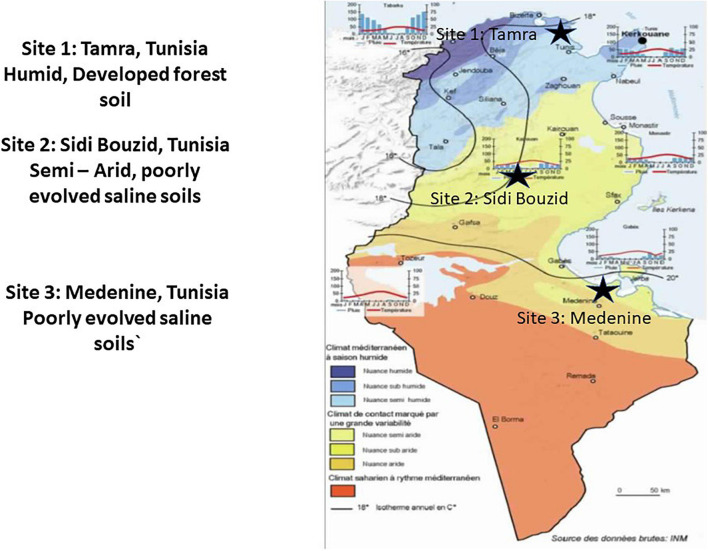
Map and schematic of amplicon survey sampling design: For each sample type from each site, 3 replicates were sampled. Modified from Hénia (2008).

Samples of *Casuarina glauca* root nodules, rhizosphere, and bulk soils. Samples were collected in February 2015 from three sites in Tunisia that represented different bioclimatic regions (Figure 1). Sample sites were in Tamra (37°3′26″ N 9°14′17″ E), Sidi Bouzid (35°2′ 7.58″ N 9° 29′2.18″ E), and Medenine (33°21′17′′ N 10°30′19′′ E), representing Mediterranean-humid, semi-arid and arid bioclimatic regions, respectively. Three to four root nodules were used for each replicate and three replicate samples were taken for each site. For the soil and rhizosphere samples, triplicate samples of 5 g soil were collected from each site. Bulk soil was soil that was not in contact with the root system of any plant. Rhizosphere soil was collected by removing organic litter and 15 cm of the surrounding soil from around the *C. glauca* roots. Soil that was attached to roots after removal of the surrounding soil was collected. The samples were placed in sterile plastic tubes and stored at −20°C until analysis. DNA extractions were performed within 24 h of sampling.

### DNA Extractions

For the rhizosphere and bulk soil samples, genomic DNA (gDNA) was extracted from 0.5 g of soil using the Qiagen DNA extraction kit as described previously (Ghodhbane-Gtari et al., 2010, 2014). For the *C. glauca* root nodule samples, three to four nodules were used per gDNA extraction and nodules were surface sterilized as described previously (Ghodhbane-Gtari et al., 2019). Root nodules were thoroughly rinsed with water. Surface sterilization conducted by shaking in 30% (v/v) H_2_O_2_ for 30 min. Sterilized nodules were rinsed thoroughly several times with sterile water. The final wash liquid was tested for microbial growth and only nodules resulting in a sterile final washing liquid were further considered for DNA extraction. The nodule was then aseptically powdered in liquid nitrogen using a sterile mortar and the resulting powder was used for DNA extraction using Qiagen DNA Plant Mini-kit following the manufacturer’s protocol.

### Amplicon and Whole Genome Shotgun Sequencing

To investigate the prokaryotic community profiles of different DNA extracted, sequences corresponding to the V4 hypervariable region the 16S subunit rRNA gene were amplified by PCR using the Earth Microbiome Project 515F/806R universal primers under conditions described previously (Caporaso et al., 2012). For each sample, triplicate amplifications were performed. The triplicates were pooled, and all samples were normalized to equal DNA concentrations. Paired-end sequencing of the amplification product was performed using the Illumina HiSeq 2500 platform at the Hubbard Center for Genome Studies (University of New Hampshire, Durham, NH, United States). Read lengths of 250 bp were obtained and processed as described below.

To generate metagenomes for the *C. glauca* root nodule samples, whole genome shotgun sequencing was performed. Sequencing libraries for the samples were prepared using the Illumina NextEra Library Preparation protocol according to the manufacturer’s instructions and were sequenced on an Illumina HiSeq 2500. The same triplicate nodule DNA extractions were used for both the metabarcoding and metagenome shotgun sequencing methods, and metagenome libraries for both methods were sequenced at 150 bp read lengths at the Hubbard Center for Genome Studies (University of New Hampshire, Durham, NH, United States).

### Amplicon Sequence Data Processing

The 16S rRNA gene amplicon sequences for each sample were initially processed with Quantitative Insights into Microbial Ecology (QIIME) pipeline (Caporaso et al., 2010b). Paired-end reads were joined using Fastq-join (Aronesty, 2011). Paired-end reads were truncated from positions where nucleotides received a Phred score of 20 or less. Reads with more than three consecutive low-quality nucleotides were discarded. Reads with less than 75% high-quality base-call scores were also discarded. This processing resulted in a total of 176,319 reads with an average length of 253 base pairs.

The reads were clustered as described in the open reference-based protocol provided in QIIME (version 1.9.0-dev) (Rideout et al., 2014). OTUs were clustered by UCLUST, using a 97% percent identity threshold to define an OTU (Edgar, 2010). Taxonomy was assigned to each OTU cluster using the UCLUST consensus taxonomy assigner by aligning the centroid sequence of each cluster to the Green Genes 13_8 rRNA database with PyNAST (DeSantis et al., 2006; Caporaso et al., 2010a; Edgar, 2010). OTUs that were unable to be aligned to the Green Genes 13_8 database were excluded from downstream analysis. OTUs that only contained one sequence (singletons) were also excluded from downstream analysis. The resulting OTU tables were filtered to exclude all unassigned sequences, and sequences that assigned to mitochondrial or chloroplast taxonomy.

### Data Normalization

The OTU table resulting from the process above was rarified to an equal sampling depth based on the smallest library size (1,762 reads) by random sub-sampling of all samples. Alternatively, Cumulative Sum Scaling (CSS), available through the metagenomeSeq R package and facilitated through QIIME, was used to normalize the OTU table (Paulson et al., 2013). Because choice of normalization method has been shown to effect downstream analysis (McMurdie and Holmes, 2014), both methods were used to compare results of normalization techniques. For Alpha diversity analysis, the original OTU table was converted to closed-reference format and corrected for variation in 16S rRNA copy number using the online Galaxy server version of PICRUSt (Langille et al., 2013). This correction was also performed on CSS normalized OTU tables and raw OTU tables in order to assess effects on ordination results.

### Amplicon Sequence Data Analysis

Alpha diversity for each sample was calculated based on the Shannon Diversity Index (Shannon, 1948) and significant differences in diversity based on sample type (Nodule, Rhizosphere, or Soil) were determined using ANOVA and the *t*-test in Microsoft Excel. Alpha diversity was also calculated in the similar manner for nodule samples from individual sampling locations. Community beta diversity among samples was measured by the UniFrac distance (Lozupone and Knight, 2005). Both the weighted (quantitative) and unweighted (qualitative) versions of the distance metric were used. Both of these metrics can yield different but complementary results from the same dataset (Lozupone et al., 2007). Distance matrices produced using these metrics were ordinated using Principle Coordinates Analysis (PCoA). Ordinations were created for distance matrices produced by CSS (for both 16S copy number corrected and not corrected), for raw OTU tables with 16S operon copy number correction and for evenly rarefied OTU tables.

Jackknifing, which is the repeated subsampling of a dataset, was used to determine the confidence level in Beta diversity analysis. This technique was performed by randomly subsampling 100 sequences per sample from the rarefied OTU table. For each subsampling, an Unweighted Pair Group Method with Arithmetic Mean (UPGMA) tree was created using the unweighted UniFrac distance matric between samples, based on those 100 randomly subsampled reads. This process was repeated 100 times. A consensus tree was built from the 100 trees with jackknife values for each node representing the number of times, out of the 100 jackknifed trees, that the consensus configuration was observed.

Nodule samples were analyzed by two-way cluster analysis to investigate the difference in nodule community structure across the environmental gradient in the sampling design. An OTU table was constructed that was corrected for 16S operon copy number abundance by PICRUSt and summarized to relative abundance at the genus level. The samples were grouped by hierarchical clustering using the average linkage method. The Bray-Curtis distance between each sample based on taxonomic composition was used for sample clustering. The taxa were clustered by their abundance patterns across samples using the Bray-Curtis distance between metric. Clustering was performed using the data for all genera present. However, for visualization purposes, only taxa that made up greater than 1% of the community were displayed.

Multi-Response Permutation Procedures (MRPP) were performed to determine if there were significant differences in phylogenetic distances based on the sample type (Nodule, Soil, or Rhizosphere). MRPP analysis was performed in R (Version 3.2.1) using the Vegan Library (Oksanen et al., 2016). MRPP To determine if nodules formed a statistically significant group based on the weighted and unweighted UniFrac distance matrices, the samples were partitioned into two categories, nodule (*n* = 9) and non-nodule (*n* = 18). Significance was calculated by permutation of the distance matrices 999 times to determine if the groups had within-group distances that were different to what would be expected by chance. All MRPP analysis was performed on the weighted and unweighted UniFrac distance matrices produced by the different normalization methods described previously (Lozupone et al., 2007; McMurdie and Holmes, 2014).

Soil and Rhizosphere samples were analyzed to test the hypothesis that *C. glauca* maintains a unique microbial community within the rhizosphere at the different sampling locations. Soil (*n* = 9) and Rhizosphere (*n* = 9) samples from all sites were pooled together and analyzed independently of nodule samples in the same fashion as described above. To determine if there were significant groupings between soil and rhizosphere by climate type, the soil and rhizosphere samples were grouped into a humid group (*n* = 6) and non-humid group (*n* = 18). MRPP was performed as above with this grouping using the unweighted UniFrac distance matrix. This metric calculates phylogenetic distance between samples based off of the presence or absence of taxa and is thus useful for identifying differences between samples that come from environments that host distinct communities.

### Metagenome Assembly and Analysis

#### Sequencing Reads Preparation and Assembly

Trimmomatic was used to remove Nextera adapters and filter the raw reads based on quality (Bolger et al., 2014). The following parameters were used. Three base-pairs were trimmed from the tailing and leading ends of each read. The reads were scanned with a sliding window of four base-pairs, and sections of reads were discarded when the average quality score of the base-pairs was below 15. After this process, only reads that retained a minimum length of 36 base pairs were used for downstream processes. Of these resulting reads, only reads that retained both forward and reverse pairs were used for assembly.

In order to perform differential coverage binning of draft genomes, the read sets from each of the three sampling locations were combined *in silico* and a composite co-assembly was created. SPAdes (version 3.6.0) was used to assemble the combined reads using the metagenomic setting, without error correction (Bankevich et al., 2012). The assembly process used Khmer lengths of 22, 33, and 55 to produce the final assembled contigs.

#### Binning

Metagenomic Assembled Genomes (MAGs) of individual members of the metagenomic community were binned using the mmgenome package (Albertsen et al., 2013) for R^2^ using the Rstudio IDE^3^. This package facilitates distinguishing individual genomes from metagenomes primarily by the unique coverage values of assembled sequences belonging to organisms present in multiple samples, but at different abundances. Coverage values for each contig per-site was performed by individually aligning each read-set to the co-assembled contigs using the Bowtie2 alignment tool (Version 2.2.5) (Langmead and Salzberg, 2012). The resulting Sequence Alignment Map (SAM) files were converted to binary format and sorted to the left-most coordinates on the assembled sequences and depth of coverage per-base was calculated using SAMtools (Version: 0.1.19-96b5f2294a) (Li et al., 2009). The resulting files containing coverage per-base per-contig were converted to average coverage per-contig scripts in the Mmgenome package.

Additional information for assembled contigs was generated following the workflow as described by the mmgenome package developers (Albertsen et al., 2013). Open Reading Frames (ORFs) within the assembled metagenomic contigs were predicted and translated to amino acid sequence using the metagenomic setting of Prodigal (Version 2.6.2) (Hyatt et al., 2010). The translated amino acid fasta file of predicted ORFs was searched against Hidden Markov Models (HMMs) of 111 essential prokaryotic single copy genes provided in the mmgenome package using hmmsearch (Eddy, 1998). ORFs identified as essential genes were extracted from the co-assembly using Perl scripts provided by the mmgenome package.

#### Taxonomic Classification

Taxonomic information was assigned to each ORF identified as essential gene by searching against the RefSeq protein database using BlastP with an *E* value cutoff of 1e^–5^, with five maximum target sequences. The resulting BlastP output in XML format was uploaded into MEGAN (Version 5) (Huson et al., 2007). The Lowest Common Ancestor (LCA) algorithm was used to make a consensus taxonomic assignment for each ORF based off of the top five BlastP hits using the following settings: Top Percent = 5, Minimum Support = 1, Rank = Species. The resulting file was parsed using the hmm.majority.vote.pl script to derive a consensus taxonomic assignment for each assembled contig based off of the taxonomy of the ORFs that it contains.

The coverage and taxonomic information produced above was loaded into the Rstudio environment along with the composite metagenomic co-assembly using BioStrings^4^. The GC content and tetranucleotide frequency distribution of each assembled contig was calculated using the mmgenome R package. A principle component analysis (PCA) of normalized tetranucleotide frequencies of all contigs was performed in R using the mmgenome package. The composite co-assembly was visualized in Rstudio and binning was performed using the tools available in the mmgenome R package. The data that was produced as described above and accompanying rmarkdown files allow the reproduction of the binned draft genomes.

#### Functional Annotation

Reads from each site were assembled individually using SPAdes-3.6.0 using the metagenomic setting (Bankevich et al., 2012). The resulting assembled contigs were annotated using the Prokka annotation pipeline using the metagenomic settings (Seemann, 2014).

### Calculation of Coverage and Relative Abundance

Coverage profiles of individual genomes with PCR duplicates removed were calculated to compare genome coverages at different sites. Reads were aligned to the metagenome assembly using the Bowtie2 alignment tool (Version 2.2.5) (Langmead and Salzberg, 2012). The SAM file resulting from alignment with Bowtie2 was converted to BAM format. The aligned reads were sorted to the left-most coordinate on the indexed metagenome assembly using SAMtools (Version: 0.1.19-96b5f2294a) (Li et al., 2009). Duplicate reads were removed using Picard Tools MarkDuplicates^5^. Coverage for the contigs was calculated from the resulting SAM file using Bedtools (v2.17.0) (Quinlan and Hall, 2010). The coverage calculations per-contig were averaged for all the contigs within a given genome bin. The average coverage for the entire genome was normalized by the amount of reads per data set corresponding to the different sampling sites. The percentage of the given read sets that aligned to plant-derived DNA was used to normalize each genome coverage value based on the differing amounts of plant-derived DNA in the different read sets.

The read sets, with plant-derived reads removed, were aligned to the *Frankia* genome using Bowtie2. The percentage of the reads that aligned to the *Frankia* genome bin were recorded from the standard output statistics in order to compare the relative abundance of *Frankia* in the shotgun data in comparison to the replicated amplicon data.

### Mining of Metagenome for Plant-Growth-Promoting Genes and Secondary Metabolites

The Genome Feature File (GFF) produced by Prokka was parsed using a custom python script to evaluate the presence and taxonomy of plant-growth-promoting genes and genes involved in cellulose, chitin, and pectin degradation within the nodule community. Genes known for plant auxin production (Indole acetic acid and Phenyl acetic acid), phosphate solubilization (alkaline phosphatases and phytases), ACC Deaminase activity and degradation of chitin, cellulose and pectin were identified for each of the three data sets. The presence of these enzymes was identified by parsing the GFF file for the Enzyme Commission (EC) number assigned to a given ORF by the Prokka pipeline. Enzymes that were identified by their EC numbers were annotated by aligning the amino acid sequence of the entry against the RefSeq protein database using BlastP with an *E* value cutoff of 1e^–5^ and a maximum output of five entries. Cladograms of the BlastP output were constructed using the Lowest Common Ancestor (LCA) algorithm in MEGAN (Version 5.0) using the following settings: Top Percent = 5, Minimum Support = 1, Rank = Genus. A summary of the EC numbers used in this analysis is in Supplementary Tables 1–3. The antiSMASH pipeline (version 3.0) was used to identify secondary metabolites in the binned genomes (Weber et al., 2015). antiSMASH was run using the metagenomic setting to improve gene prediction for highly fragmented metagenomic assemblies.

## Results

### Diversity of the *Casuarina* Nodule Community and Surrounding Soil

**FIGURE 2:**
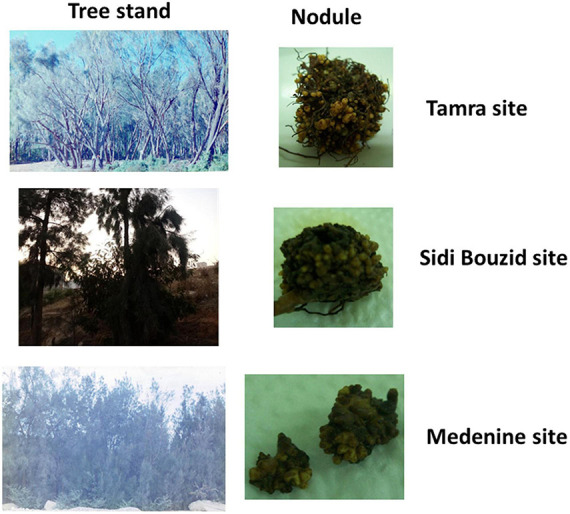
Photograph of *Casuarina* tree stands at the sampling and representative nodules for those trees.

Three distinct sites in Tunisia (Figure 1) were chosen to assess the effect of environmental conditions on the microbial community structure and diversity for *Casuarina* nodules, rhizosphere and surrounding soil. These sites represented a humid well-developed forest at Tamra, a semi-arid, poorly evolved saline soils of Sidi Bouzid and an arid, poorly evolved saline soils of Medenine. Figure 2 shows representative *Casuarina* stands from each of these sites and nodules from those stands. There were no obvious differences in nodule shape or size among the three sites and the plants exhibited the same level of general health.

Preprocessing of the 16S rRNA gene amplicon sequences resulted in a total of 7,855 OTUs from the 149,879 paired reads. Individual library sizes ranged from 1,762 to 11,542 reads per sample, with a mean of 5,551 reads per sample. Rarefaction plots were performed in order to estimate the completeness of community sampling at the sequencing depth performed. The rarefaction plots for average observed OTUs per sample type is shown in Supplementary Figure 1. These plots show that the nodule samples were sequenced close to saturation, while increased sequencing depth could improve the documentation of rare OTUs for rhizosphere and soil samples.

### Alpha Diversity

The Shannon Diversity Index (SDI) was used to measure alpha diversity. This metric was measured on the relative abundance after correction for 16S operon copy number. Diversity measures were grouped by sample type and are as follows; Nodules: 4.47 ± 2.29 (SD), Rhizosphere: 9.33 ± 0.233 (SD) and Soil: 9.52 ± 0.69 (SD), displayed in Supplementary Figure 2A. The alpha diversity was significantly less for nodules than for rhizosphere and soil samples (Students *t*-test, *p* = 0.00001). Alpha diversity was calculated for the nodule samples at the three different samples locations. Nodules from Tamra had an average SDI of 2.28 (SD = 0.584), nodules from Sidi Bouzid had an average SDI of 7.32 (SD = 0.389) and nodules from Medenine had an SDI of 3.82 (SD = 0.394) (Supplementary Figure 2B).

**FIGURE 3:**
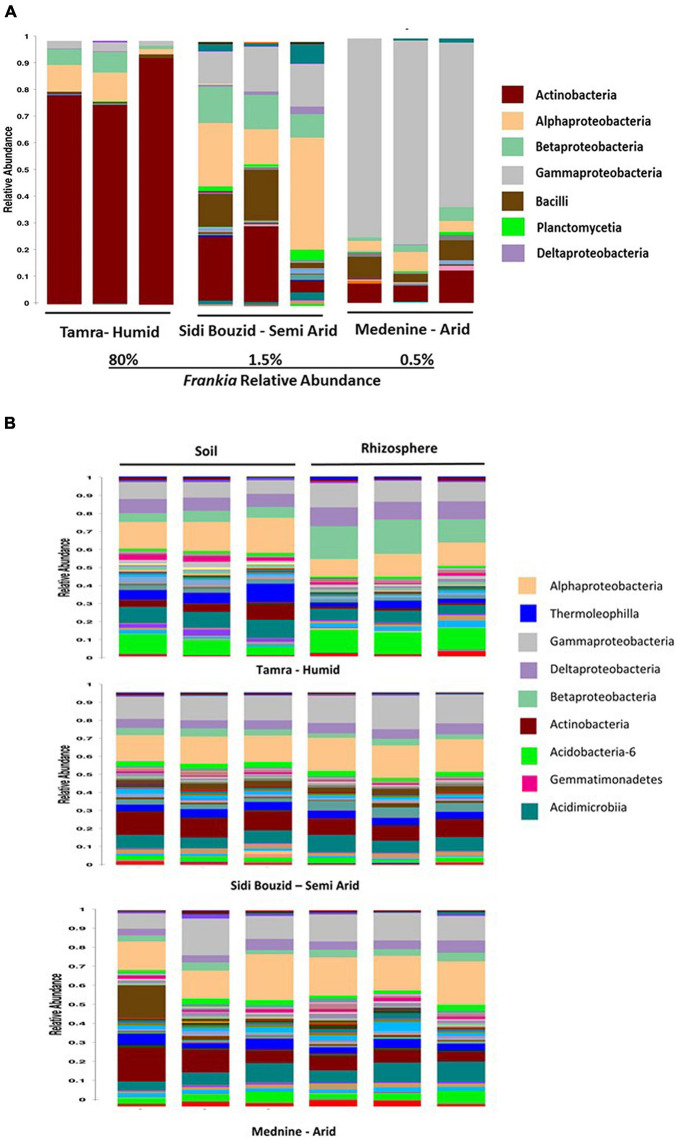
Class level taxonomic summary of nodule, Rhizosphere and soil samples. **(A)** Nodules grouped by site. The relative abundance of the nodule samples was summarized at the class level after corrected for 16S operon copy number variation. The humid and arid samples are significantly less diverse than the semi-arid samples, as can be seen in **(A)**. This can be seen here, as the humid site is dominated by Actinobacteria (the genus *Frankia*), and the arid site is dominated by *Acinetobacter* and *Pseudomonas.* **(B)** Rhizosphere and soil samples grouped by sampling site. These charts show relative abundance of taxa at the class level after correction for 16S operon copy number.

After correction for 16S operon copy number, the relative abundance of taxa present in the root nodules was analyzed. At the humid sampling site, the relative abundance of *Frankia* was an average of 80% of the nodule community. This prevalence dropped to 1.5% and 0.5% and the semi-arid and arid sampling sites, respectively. These data are like the shotgun sequencing data (Supplementary Figure 3), where reads mapping to the binned *Frankia* genome comprised 60% of the humid site read set and that level dropped to 1.71% and 1.63% for the semi-arid and arid sampling sites, respectively. Supplementary Figure 3 shows the relative abundance of *Frankia* at the three sampling sites using the shotgun and amplicon data. Figure 3A gives a taxonomic summary of the nodule samples at the class level. The taxonomic summary of the rhizosphere and bulk soils is shown in Figure 3B.

### Beta Diversity

**FIGURE 4:**
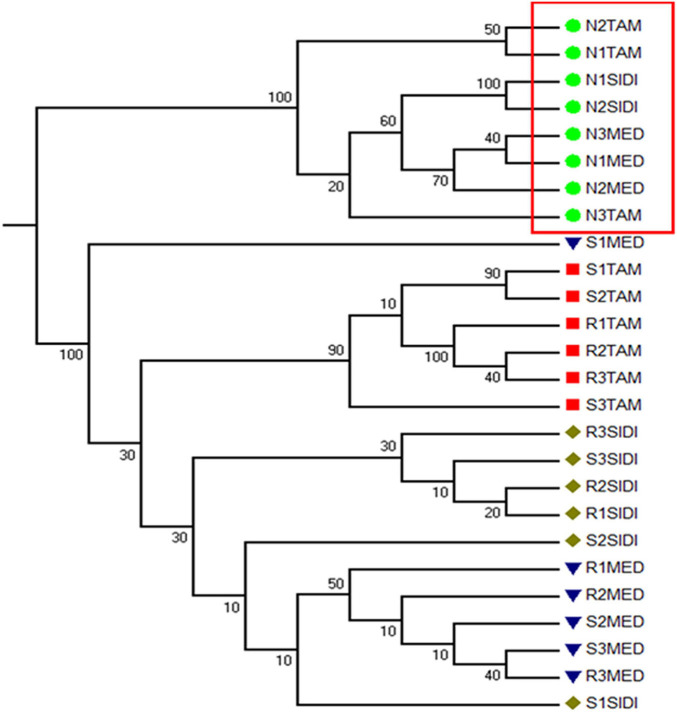
Unweighted pair group method with Arithmetic Mean (UPGMA) Consensus Tree. The tree was constructed from the rarefied unweighted UniFrac distance Matrix. The consensus tree was built by creating 100 trees from 100 randomly subsampled reads from each sample. The numbers on the nodes represent the percentage of the 100 trees that conformed to this configuration. The red box outlines that all nodule samples form one cluster on the tree. The other clades on the tree show that the soil and rhizosphere communities cluster mostly by sampling site.

Beta diversity was analyzed by measuring the weighted and unweighted UniFrac distances (Lozupone and Knight, 2005). An Unweighted Pair Ground Method with Arithmetic Mean (UPGMA) consensus tree was built from the unweighted UniFrac distance matrix (Figure 4). Numbers on the nodes of the tree represent the percentage of trees out of 100 bootstrapped trees that had the configuration of the final consensus tree. The grouping on the tree shows that nodule samples from all sites form one clade when analyzed by the presence or absence of taxa in the samples, after removing one outlier (Sample ID: N3SIDI). This result shows that although the relative abundance of taxa within the nodule differed from sampling location (Figure 3 and Supplementary Figure 3), the nodules are distinct based on what taxa they host when compared to the soil and rhizosphere samples. The rhizosphere and soil primarily cluster based off the sampling location, this trend is also evident in ordinations of the data using the unweighted UniFrac distance metric (Supplementary Figure 4).

**FIGURE 5:**
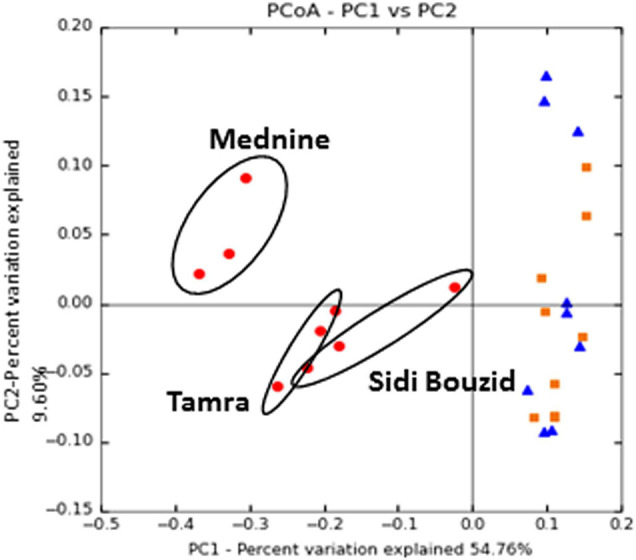
Principal coordinates ordination of CSS normalized UniFrac distance matrices: Ordinations where normalized using Cumulative Sum Scaling. Ordinations was made using the weighted UniFrac distance. Symbols represent: nodules (•), rhizosphere (■), and soil (▲). For all ordinations MRPP showed a statistically significant grouping of the nodule samples, as outlined in Supplementary Table 4.

The beta diversity distance matrices were ordinated using PCoA. Distance matrices were constructed using the weighted and unweighted UniFrac distances on OTU tables that had been normalized by rarefaction, a raw OTU table with ribosomal operon copy number correction and by cumulative sum-scaling (with and without ribosomal operon copy number correction). PCoA ordinations of these distance matrixes can be seen in Figure 5 and Supplementary Figures 4, 5. For the sake of clarity, Figure 5 represents weighted UniFrac distances normalized by cumulative sum-scaling without ribosomal operon copy number correction. All of the methods gave similar results (Supplementary Figure 4). In all ordinations, nodule samples are separated from the soil and rhizosphere samples along the first Principal Coordinate, which explains the most phylogenetic variation in the data. Multi-response Permutation Procedures (MRPP) agreed with the results seen in the UPGMA tree, with significant grouping of nodules for every normalization type using the both forms of the UniFrac distance metric. Supplementary Table 4 summarizes the chance-corrected results of the MRPP analysis. The A Statistics, the chance-corrected within-group agreement, is a value between 1 and 0 that describes the within-group heterogeneity of a given group. If all samples in a group are identical A = 1, if the heterogeneity in a group is equal to what would be expected by chance A = 0 (Mielke and Kenneth, 2007).

### Nodule Analysis

The results of the UPGMA Tree (Figure 4) and the PCoA ordinations (Figure 5 and Supplementary Figures 4, 5) show that nodules host a distinct prokaryotic community compared to the soil and rhizosphere samples. Ordination and MRPP results show that this grouping is statistically significant whether the unweighted UniFrac or weighted UniFrac is used.

Nodules form a distinct group based on the taxa they host, but the relative composition of this group of taxa changes drastically along the environmental gradient sampled (Figure 3A). In Figure 5, the ordinations show the grouping of nodule samples by location along the second principal coordinate, which explains the second largest amount of taxonomic variation in the dataset.

### Rhizosphere and Soil Analysis

To understand differences in the rhizosphere and soil communities across the sampling sites, these samples were analyzed separately from the nodule samples. Rhizosphere soils from all sites were analyzed as one sample category to test the hypothesis that *C. glauca* hosts a distinct microbial community within the rhizosphere across environmental gradients. When soil and rhizosphere samples from all sites were grouped together by type, the MRPP results were inconsistent in their significance, depending on the OTU table normalization strategy. A summary of these results is found in Supplementary Table 5. Statistical significance (*P* < 0.05) was only observed with the evenly rarefied OTU table and the raw OUT table that corrected for 16S operon copy number Variation. Ordinations of these data (Supplementary Figures 4, 5) show that the rhizosphere and soil samples formed two clusters only on the raw OTU table and the evenly rarefied OTU table. CSS normalization produces different results with the soil and rhizosphere samples not forming distinct groups.

The ordinations using the unweighted UniFrac distance, which considers the presence and absence of taxa, show that soil and rhizosphere samples clustered based on climate. When the data is ordinated using this metric, two distinct clusters of rhizosphere and soil samples formed Supplementary Figure 6. These clusters correspond to the humid sampling site (Tamra) and the two arid sampling sites (Sidi Bouzid and Medenine). For all normalization types, a significant grouping was identified by MRPP (Supplementary Table 6), showing that the environmental conditions significantly change the taxa present between the humid and arid soils that were sampled. A taxonomic summary of these samples at the class level are presented in Figure 3B.

### Shotgun Metagenomic Sequencing

Quality filtering of shotgun metagenomic reads resulted in a total of 83,027,118 paired end reads. The library for Sites 1, 2, and 3 were comprised of 33,051,352, 34,239,886, and 15,735,880 reads, respectively. *In silico* co*-*assembly of the reads resulted in an assembly contained 3,153,711 contigs totaling 56,964,841 base pairs in length. The statistics for the assembly produced by Quast (Gurevich et al., 2013) are outlined in Supplementary Table 7.

### Metagenomic Assembled Genomes Reconstructed Using Differential Coverage Binning

**FIGURE 6:**
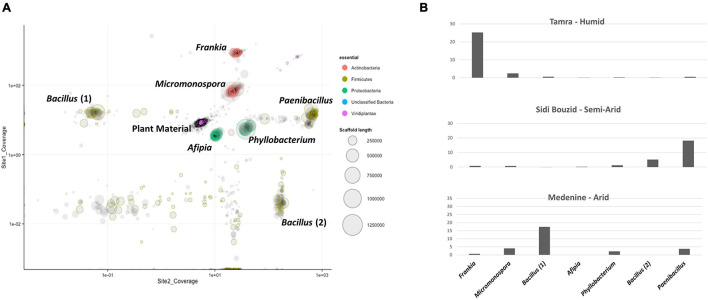
**(A)** Plot of metagenomic contigs larger than 5 kbp produced by the mmgenome R Package. The seven dominant genomes that were binned using the mmgenome R package are labeled with their annotated genus. The purple and black cluster in the center of the image are contigs derived from *C. glauca* DNA. **(B)** Normalized genome coverage of genome bins. Reads from each of the three sampling sites were aligned to the co-assembled contigs. SAMtools was used to sort and index the coordinates of the aligned reads and Picard MarkDuplicates was used to remove duplicate reads with identical coordinates. Bedtools was then used to calculate coverage per contig per site. Coverage values were normalized by millions of reads per data set and the relative amount of plant DNA that comprised each data set.

Seven predominant genomes were binned using the mmgenome R package. A plot of the metagenomic assembly can be seen in Figure 6A. The binned genomes were identified being in the genera *Frankia*, *Micromonospora*, *Bacillus* (two isolates), *Phyllobacterium*, *Paenibacillus*, and *Afipia.*

Two of the binned genomes annotated to the phylum Actinobacteria. The first genome bin contained 4,111 ORFs that annotated to the genus *Frankia*. The annotations for the ORFs in this bin had top hits on many of the *Frankia* clade 1c *Casuarina* infective strains. The most top annotations that hit on a single sequenced strain was 775 of the 4,111 ORFs annotated to *Frankia* sp. BMG5.23, a salt-tolerant Tunisian isolate (Ghodhbane-Gtari et al., 2014). Reads mapping to the binned *Frankia* draft genome comprised 60% of the reads in from the humid sampling site, Tamra. Of the reads from semiarid Sidi Bouzid and arid Gabes, 1.7% and 1.6% of the reads mapped to the *Frankia* genome bin, respectively. Genome bin two resulted in an assembly that contained 6,522 ORFs with 6,053 annotating to the genus *Micromonospora.* The ORFs that annotated to *Micromonospora* were mainly comprised of annotations to *Micromonospora purpureochromogenes*, with 4,565 ORFs. Three genomes that were binned annotated to the phylum Firmicutes. The first contained 6,268 ORFs with 5,866 of them annotating to the genus *Paenibacillus.* The second Firmicutes genome bin contained 4,275 ORFs, 4,060 of which annotated to the genus *Bacillus.* Of those ORFs annotating to the genus *Bacillus*, 3,416 of them annotated to *B. simplex.* The last Firmicutes genome had 4,215 ORFs that also annotated to the genus *Bacillus* with 3,313 of them annotating to *B. aquimaris* and 569 annotating to *B. vietnamensis*. Two of the binned genomes annotated to the phylum proteobacteria. The first binned genome contained 4,370 ORFs that annotated to the genus *Afipia.* The second proteobacteria genome bin contained 3,068 ORFs that annotated to the genus *Phyllobacterium*. General genome characteristics of the binned genomes are summarized in Table 1.

**TABLE 1:** General assembly characteristics of each binned genome.

Isolate genus	Closest annotation	Assembly length	Open reading frames	GC%
*Frankia*	*Frankia* sp. BMG5.23	4.92 Mbp	4,111	70.25%
*Micromonospora*	*M. purpureochromogenes*	7.28 Mbp	6,522	72.26%
*Bacillus* (1)	*B. simplex*	4.64 Mbp	4,060	40.0%
*Bacillus* (2)	*B. aquimaris/vietnamensis*	4.7 Mbp	4,215	41.53%
*Phyllobacterium*	*Phyllobacterium* sp. *UNC302MFCol5.2*	4.8 Mbp	3,068	59.42%
*Afipia*	*A. broomeae*	5.3 Mbp	4,370	61.64%
*Paenibacillus*	*Paenibacillus* sp. HGF5	7.0 Mbp	5,866	49.56%

*The assembly length and GC content were calculated by Quast. Open reading frames were predicted using Prodigal. Closest annotations were determined after aligning all ORFs to the RefSeq protein database.*

### Coverage Values for Genome Bins

Genome coverage for binned genomes was calculated in order to compare coverage patterns across sites to the replicated 16S rRNA gene amplicon survey. The only binned genome which followed the same patterns as the replicated analysis was the genome bin that annotated as a member of *Frankia* (Figure 6A) compared to the relative abundance of *Frankia* between the two datasets. For those calculations, reads that aligned to plant-derived DNA was first removed. The resulting read sets for each site were aligned to the reassembled *Frankia* genome bin using Bowtie2. The percentage of those reads that aligned to the genome bin was recorded. The *Frankia* genome bin was the dominant member of the humid site, but coverage values were much lower for the semi-arid and arid sampling sites (Figure 6B).

### Secondary Metabolites Identified in Binned Genomes

The MAGs reconstructed from the metagenomic dataset were analyzed for the presence of secondary metabolite biosynthetic pathways using AnitSMASH (Weber et al., 2015). The secondary metabolite biosynthetic clusters that were identified in the binned genomes are outlined in Table 2. Predicted biosynthetic clusters from the binned genomes were predicted to produce siderophores, terpenes, non-ribosomal peptide synthases and other secondary metabolites. The genome *Frankia* and *Micromonospora* genome bins contained the most secondary metabolite biosynthetic clusters about 25 for each bin genome, although only clusters which have a predicted product are listed here.

**TABLE 2:** Secondary metabolite biosynthetic gene clusters identified each genome bin by antiSMASH.

Bin	Genus	Type	Most similar known cluster	Percent identity
	*Frankia*	Terpene	Sioxanthin	60%
		Type-Two PKS	Frankiamicin	100%
		Lassopeptide-Type-One PKS	Maklamicin	13%
		Terpene	Hopene	38%
		Other	Streptolydigin	7%
		NRPS	Triostin	16%
		Type-One PKS	Divergolide	13%
		Terpene	Collismycin A	7%
		Type-Two PKS	Medermycin	30%
		Ketide Synthase-Butyrolactone	Abyssomicin	10%
		Other	A47934	10%
		Type-Three Polyketide Synthase	Feglymycin	52%
	*Micromonospora*			
		T1PKS	Maklamicin	19%
		NRPS-T1PKS	Nostopeptolide	37%
		Other	Diazepinomicin	75%
		Oligosaccharide-PKS-Terpene	Brasilicardin A	54%
		Siderophore	Desferrioxamine B	80%
		T2PKS	Xantholipin	16%
		Terpene	Nocathiacin	4%
		Lantipeptide	Pentalenolactone	15%
		NRPS	Friulimicin	12%
		T3PKS	Alkyl-O-Dihydrogeranyl-Methoxyhydroquinone	71%
		Nrps-T1pks-Lantipeptide	Bleomycin	12%
		Terpene	Sioxanthin	80%
		Nrps-Lantipeptide-T1pks-Others	Naphthyridinomycin	14%
		T1PKS	Calicheamicin	13%
		Terpene-Bacteriocin	Lymphostin	30%
		T1pks-Nrps	Rifamycin	35%
		T1pks	Leucanicidin	100%
	*Paenibacillus*	Ectoine	Ectoine	75%
		Nrps	Bacillibactin	53%
		Trans-At Pks	Bacillaene	21%
	*Bacillus (1)*	Siderophore	Desotamide	9%
		Nrps	Koranimine	87%
	*Bacillus (2)*	Terpene	Carotenoid	33%
		Siderophore		16%
	*Afipia*	Terpene	Malleobactin	11%
	*Phyllobacterium*	Nrps	Vicibactin	77%

### Functional Mining of Metagenomes

**FIGURE 7:**
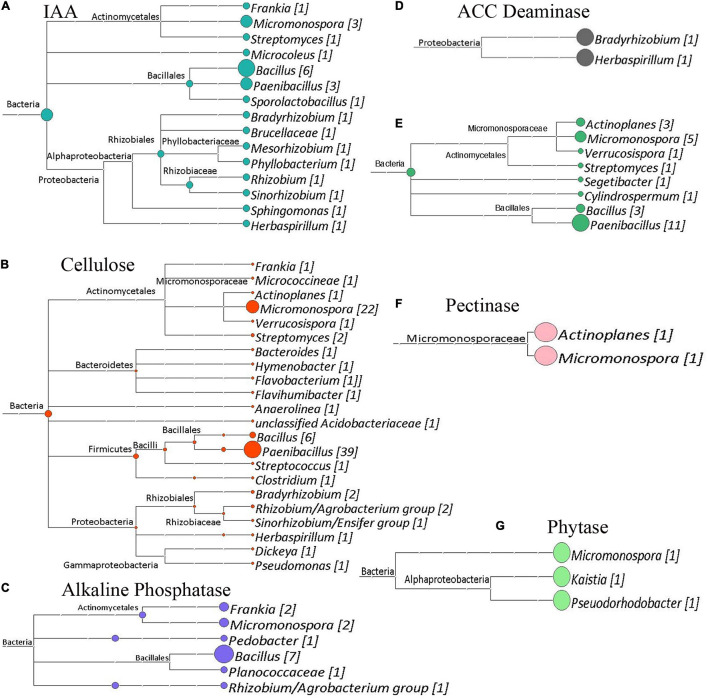
Cladograms of functional genes from Site 1 (Tamra), that were produced by the LCA Algorithm in MEGAN (Version 5). The sizes of colored circles represent that relative contribution of a taxa to the functional genes analyzed in a given cladogram. Numbers in Brackets represent the number of genes that were annotated to a given taxon for that set of genes. **(A)** Potential indole acetic acid biosynthetic genes. Total genes, 24. **(B)** Cellulose degrading genes. Total genes identified, 89. **(C)** Alkaline phosphatase genes. Total genes identified, 26. **(D)** ACC deaminase genes. Total genes Identified, 2. **(E)** Chitin degrading genes. Total genes identified, 14. **(F)** Pectinase genes. Total genes identified, 2. **(G)** Phytase genes. Total genes identified, 2.

**FIGURE 8:**
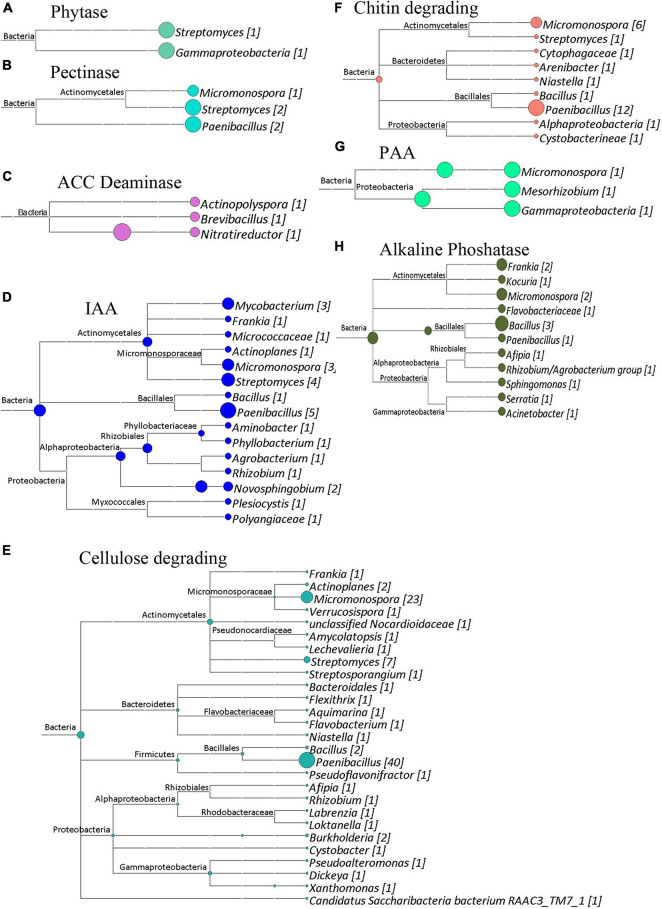
Cladograms of functional genes from Site 2 (Sidi Bouzid), that were produced by the LCA Algorithm in MEGAN (Version 5). The sizes of colored circles represent that relative contribution of a taxa to the functional genes analyzed in a given cladogram. Numbers in Brackets represent that number of genes that annotated to a given taxon for that set of genes. **(A)** Phytase genes. Total genes identified, 8. **(B)** Pectinase enzymes. Total genes identified, 5. **(C)** ACC deaminase genes. Total genes identified, 3. **(D)** Potential indole acetic acid biosynthesis genes. Total genes identified, 27. **(E)** Cellulose degrading enzymes. Total genes identified, 97. **(F)** Chitin degrading enzymes. Total genes identified, 25. **(G)** Potential phenyl acetic acid biosynthesis genes. Total genes identified, 3. **(H)** Alkaline phosphatase genes. Total genes identified, 15.

**FIGURE 9:**
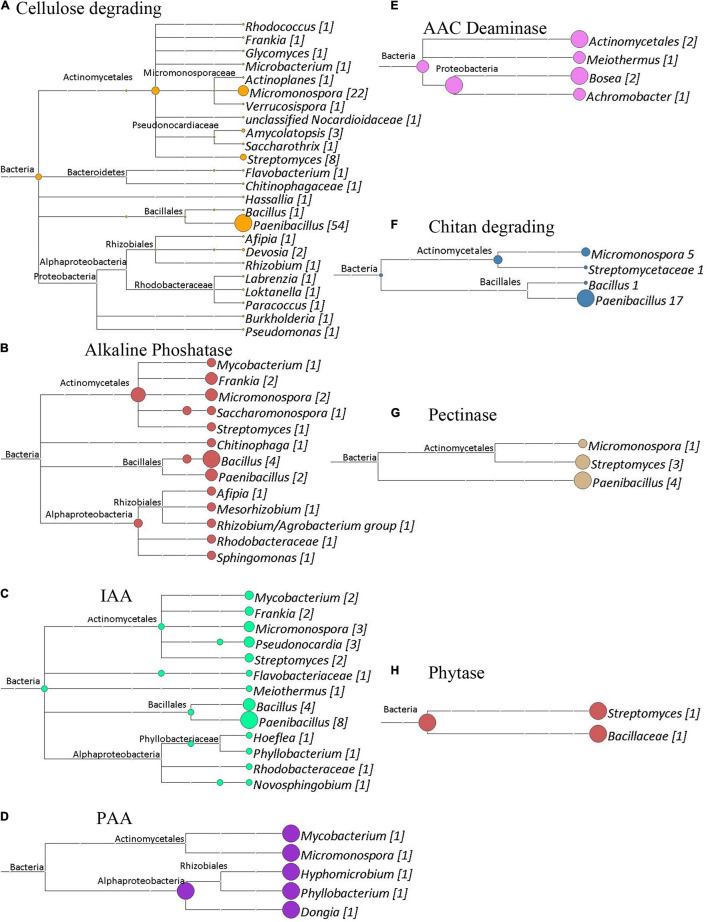
Cladograms of functional genes from Site 3 (Medenine), that were produced by the LCA Algorithm in MEGAN (Version 5). The sizes of colored circles represent that relative contribution of a taxa to the functional genes analyzed in a given cladogram. Numbers in Brackets represent that actual number of genes that annotated to a given taxon for that set of genes. **(A)** Cellulose degrading genes. Total genes identified, 108. **(B)** Alkaline phosphatase genes. Total genes identified, 19. **(C)** Potential indole acetic acid biosynthetic genes. Total genes identified, 30. **(D)** Potential phenyl acetic acid biosynthesis genes. Total genes identified, 5. **(E)** ACC deaminase genes. Total genes identified, 6. **(F)** Chitin degrading genes. Total genes identified, 24. **(G)** Pectinase genes. Total genes identified, 8. **(H)** Phytase genes. Total genes identified, 2.

The shotgun metagenomic data was mined for genes of interest with potential functional importance to the actinorhizal symbiosis and *C. glauca* health. Genes potentially responsible for plant–growth-promoting traits were mined from GFF files produced by the Prokka Annotation pipeline (Seemann, 2014). Figures 7–9 show the results of this analysis as outlined by site. Cladograms for the taxonomy of the genes identified are presented. The number of genes for each function is reported in each figure. The sampling sites 1, 2, and 3 had 159, 183, and 202 genes of interest, respectively. Since shotgun sequencing was not performed with replicates, these results are presented qualitatively as presence or absence data.

## Discussion

### Prominent Microbial Groups of Nodules, Rhizosphere, and Soil Associated to *Casuarina glauca*

The 16S rRNA gene amplicon analysis of the taxonomic community in nodules showed that they were largely dominated by Actinobacteria, Alphaproteobacteria, and Gammaproteobacteria (Figure 3). The remaining sequences matched with Bacilli, Planctomycetia and Deltaproteobacteria. A remarkable change in microbial community dominance was observed across climate stages. In the humid climate zone, represented by Tamra site, Actinobacteria was the most dominant class, while the semiarid (Sidi Bouzid) and arid (Medenine) environments had Alphaproteobacteria and Gammaproteobacteria, respectively, as the most prominent class. This result was surprising because *Frankia* is Actinobacteria and would be expected to dominate the nodule environment.

However, the relative abundance of *Frankia was* drastically affected by the environmental gradient. Nodule occupancy was highest (80%) in plants at the Tamra (humid zone); but was drastically reduced in nodules from the semi-arid Sidi Bouzid (1.5%) and arid Medenine (0.5%). One possible explanation of this phenomenon is that the physical and chemical characteristics of semi-arid and arid environments are not beneficial for the maintenance of the actinorhizal symbiosis or *Frankia* persistence. Soils in the semi-arid and arid regions of Tunisia are poorly evolved calcareous soils (Mtimet, 2001). In these soils, phosphorous is known to be immobilized by formation of complexes with calcium cations (Tunesi et al., 1999). Low phosphorus levels decrease the frequency of *C. cunninghamiana* nodulation by *Frankia* (Yang, 1995) and nodule weight of *Frankia-C. equisetifolia* (Sanginga et al., 1989). Similarly, drying and heat are known to decrease the ability of some *Frankia* strains to infect *C. equisetifolia* (Sayed, 2011). Dryness, high temperature and low levels of available phosphorous are characteristics of soils in arid environments and these factors could be influencing the low abundance and absence of *Frankia* in nodules at these environments. Detecting *Casuarina* infective clades of *Frankia* through plant-trapping assays have proven difficult (Gtari et al., 2002, 2007) and the scarcity of detection of *Casuarinaceae-*infective *Frankia* outside of the plants native range has been demonstrated (Mirza et al., 2007). One possible explanation is that *Casuarina-*infective *Frankia* are more persistent in humid environments that are more similar to the riparian habitat that is preferred by *Casuarina* within their host range. These results are congruent with the need for intentional inoculation with a compatible *Frankia* strain necessary for nodulation in some land reclamation projects in Africa (Gauthier et al., 1985). However, this reasoning does not explain the absence of *Frankia* within mature nodules. The absence of detected *Frankia* in four of the nine nodule samples leads to one hypothesis that another member of the community may be responsible for the formation of nodules on *C. glauca*. *Agrobacterium rhizogenes* will induce pseudonodules on *Elaeagnus angustifolia* that are indistinguishable from *Frankia* – induced nodules (Berg et al., 1992). Our results show an OTU of genus *Agrobacterium* that was as differentially abundant in nodule samples. The genus *Agrobacterium* was present in eight of the nine nodule samples ranging from 0.01% to 2.8% relative abundance. This suggests that a member of this genus could play a role in nodule formation when *Frankia* is in low abundance. Another hypothesis is that this absence is a seasonal variation and part of the life cycle of *Frankia* under semi-arid and arid conditions. As the levels of *Frankia* decreases, other microbes could establish more dominance within the nodule. A seasonal study of the changes in community structure could address this hypothesis.

### Distinct Nodule Community Across Sampling Sites

Multi-response permutation procedures analysis found that nodule samples formed a statistically significant group compared to the rhizosphere and soil samples (Figure 5 and Supplementary Figures 4–6). Nodule samples are separated from the rhizosphere and soil samples by the first principal coordinate axis, which explains the most taxonomic information in the samples. This result is also confirmed with the jackknifed UPGMA tree built from the unweighted UniFrac distance matrix (Figure 4), which shows a distinct clade where all nodule samples clustered, despite location. Alpha diversity of the nodule samples was also significantly lower than for the rhizosphere or soil samples (Supplementary Figure 1). These observations are similar to comparative analysis of the root microbiome of the model plant *Arabidopsis thaliana*, which has shown that endophytic communities from plants growing in chemically distinct soil types are highly overlapping and less diverse than surrounding soils (Lundberg et al., 2012). The community selected by *C. glauca* was distinct from the soil and rhizosphere communities across a steep environmental gradient and was significantly less diverse, which is like the pattern of the A. *thaliana* microbiome. The similar patterns of microbiome recruitment by *C. glauca* and *A. thaliana* suggests that phylogenetically distant plant groups tend to select for a particular endophytic community across environmental gradients.

### Functions of Taxa in Nodules

From metagenome analysis, seven predominant genomes were identified by binning (Figure 6) and were assigned to six different genera: *Frankia*, *Micromonospora*, *Bacillus* (two isolates), *Phyllobacterium*, *Paenibacillus*, and *Afipia*. Reads from the humid sampling site Tamra, contains the most reads mapping to the binned *Frankia* draft genome (60%), then Sidi Bouzid (1.7%) and Medenine (1.6%). The less abundance of *Frankia* in semi-arid and arid sites can be related to the harsh conditions, dryness and poor composition of soils. Not surprisingly, the *Frankia* MAG closely matched the *Frankia* sp. strain BMG5.23 genome (Ghodhbane-Gtari et al., 2014). This strain was isolated from Tunisian soils and probably represents the local strain in this region.

By the use of the antiSMASH program (Blin et al., 2017), secondary metabolite biosynthetic pathways were predicted from the draft genomes rebuilt from the metagenomic data set (Table 2). Not surprisingly, the Actinobacteria, *Frankia* and *Micromonospora*, genomes contained the most secondary metabolite biosynthetic clusters. Actinobacteria are well known for their large assembly of secondary metabolic pathways producing a wide array of natural products.

The presence of non-*Frankia* bacteria within the nodules confirms other observations (Guillén et al., 1993; Niner et al., 1996; Solans and Vobis, 2003; Valdes et al., 2005; Trujillo et al., 2006; Solans, 2007; Ghodhbane-Gtari et al., 2010, 2014, 2019; Solans et al., 2011). In particular, the presence of *Micromonospora* is noteworthy having been identified as an endophyte of actinorhizal and leguminous plants (Trujillo et al., 2006, 2007; Solans et al., 2011) and considered a normal occupant of actinorhizal nodules (Carro et al., 2013). Two different *Bacillus* genomes were identified with one being closely related to *B. simplex* and the other being closely related to *B. aquimaris* and *B. vietnamensis*. *Bacillus simplex* is known for its adaptations to arid environments (Sikorski and Nevo, 2005) and has been shown to acts as a helper-bacteria for the symbiosis between *Pisum sativum* and *Rhizobium leguminosarum* bv. *viciae* (Schwartz et al., 2013). *Bacillus* isolates are helper-bacteria for the *Frankia-Casuarina* symbiosis (Echbab et al., 2004).

*Paenibacillus* genome revealed secondary metabolic clusters for siderophore production, ectoine, (an osmoprotectant), and bacillaene (an antibiotic). In a nursery trail, co-inoculation of *C. equisetifolia* with *Paenibacillus polymyxa* and *Glomus geosporum* results in the highest seedling quality (Muthukumar and Udaiyan, 2010), showing the potential benefits of this nodule occupant. One hypothesis is that these non-*Frankia* Actinobacteria act as helper-bacteria in the formation of the actinorhizal symbiosis (Ghodhbane-Gtari et al., 2010, 2019; Solans et al., 2011; Ghodhbane-Gtari and Tisa, 2014). The absence of *Nocardia* within these nodule communities was surprising. *Nocardia* strains (BMG51109 and BMG111209) were isolated from *Casuarina glauca* in Tunisia (Ghodhbane-Gtari et al., 2010) and shown to have plant-growth promoting properties (Ghodhbane-Gtari et al., 2019). Co-infection studies showed that *Nocardia casuarinae* strain BMG51109 plays a role as a “helper bacteria” promoting an earlier onset of nodulation (Ghodhbane-Gtari et al., 2019).

Besides generating metagenomes, the shotgun data set was functionally mined to identify potential plant-growth-promoting genes (PGPG) and to phylogenetically analyze these data (Figures 7–9) suggesting a wide berth of potential PGPG genetic capability for plant growth promotion. Besides this phylogenetic analysis, these coding regions were predicted to produce enzymes and metabolites of relevance to the benefiting plant health. All three metagenomes contained the 1-aminocyclopropane-1-carboxylate (ACC) deaminase gene. ACC deaminase plays an important role in the nodulation process of leguminous plants (Nascimento et al., 2019) and has been shown to improve plant growth under arid (Bessadok et al., 2020) or saline regions (Orozco-Mosqueda et al., 2020). Other major functions involved in nutrient recycling, suppressing phytopathogens and mineralization of organic matter were also highly abundant within all three metagenomes. The enzymes, chitinase, phytase, cellulase, pectinase and alkaline phosphatase are well known to be involved in the preservation of the plant fertility and maintaining the healthy plant growth (Neeraja et al., 2010; Maksimov et al., 2011; Jain and Singh, 2017). These hydrolytic enzymes serve as an important defense strategy to defend against phytopathogenic fungi (Lynd et al., 2002). These enzymes may serve to help the *Casuarina* to survive from fungal pathogens.

Genes for production of phytohormones were also present at all three sites in variable proportions. Genes for IAA and PAA production were significantly enriched in the semi-arid and arid climates. IAA and PAA play a key role in many aspects of plant growth and cell elongation and division (Champion et al., 2015; Sugawara et al., 2015). The increased number of IAA and PAA genes suggest that these phytohormones may alter the plant physiology to respond to these arid conditions.

## Conclusion

The microbial community of the nodules appears to be shaped by the different bioclimates found in Tunisia. Beside *Frankia* microsymbiont, the niche builder of root nodules, other bacteria may occur within. However, further work is required to understand the PGP mechanisms of the different co-inhabitants of the nodule and more diverse samples from a wide range of actinorhizal plant nodules is needed to deepen this study.

## Data Availability Statement

The datasets presented in this study can be found in online repositories. The names of the repository/repositories and accession number(s) can be found below: https://www.ncbi.nlm.nih.gov/bioproject/, PRJNA482626.

## Author Contributions

FG-G, MG, and LT conceived the study. FG-G and TD’A performed the research. FG-G, TD’A, AG, SG, MG, and LT analyzed the data. FG-G, TD’A, MG, and LT wrote the manuscript. All authors approved the manuscript.

## Conflict of Interest

The authors declare that the research was conducted in the absence of any commercial or financial relationships that could be construed as a potential conflict of interest.

## Publisher’s Note

All claims expressed in this article are solely those of the authors and do not necessarily represent those of their affiliated organizations, or those of the publisher, the editors and the reviewers. Any product that may be evaluated in this article, or claim that may be made by its manufacturer, is not guaranteed or endorsed by the publisher.

## Footnotes

1
http://fao.org

2
http://r-project.org

3
http://Rstudio.com

4
https://bioconductor.org/packages/Biostrings

5
http://broadinstitute.github.io/picard/
