# Supplementary material for: Alone Yet Not Alone: Frankia Lives Under the Same Roof With Other Bacteria in Actinorhizal Nodules
Source: Front Microbiol. 2021 Dec 2;12:749760. doi: 10.3389/fmicb.2021.749760 (PMC8674757; doi:10.3389/fmicb.2021.749760)
Supplement: Supplementary file 1 [file Data_Sheet_1.pdf]

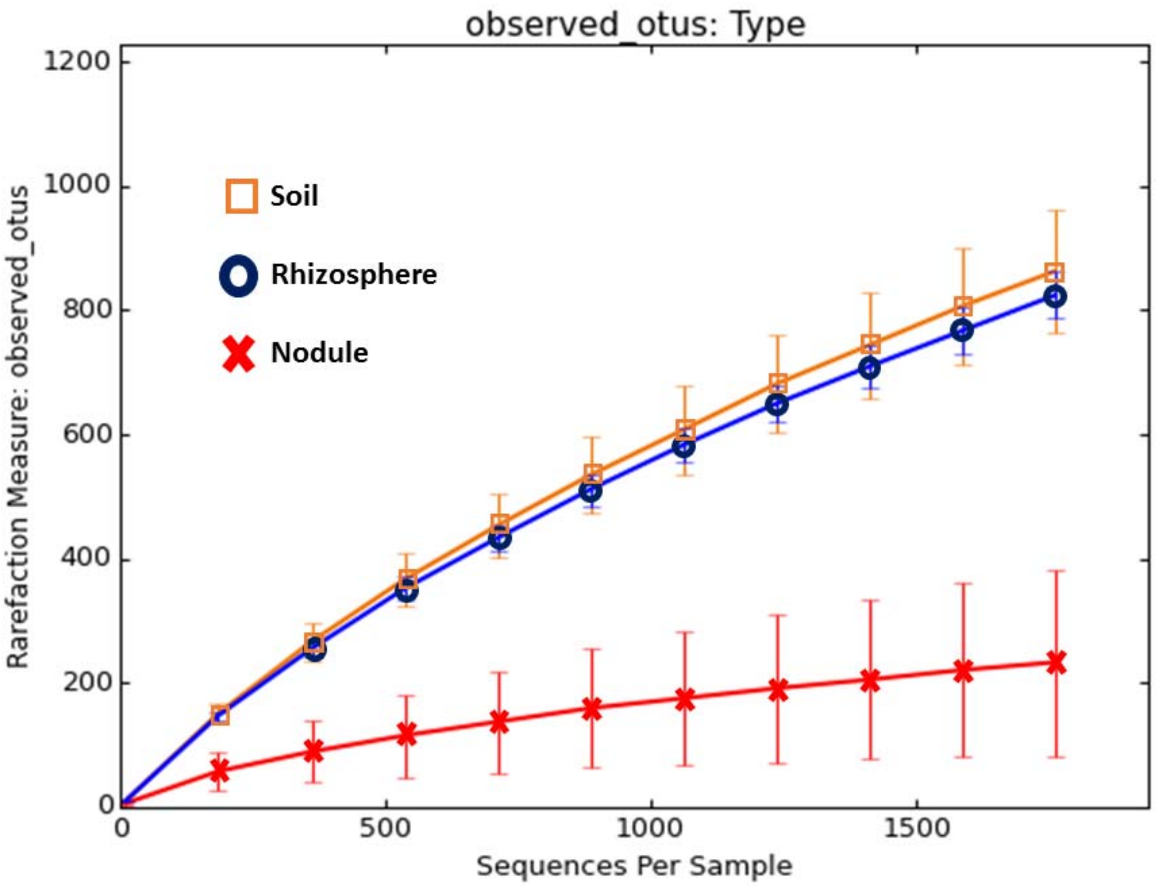

Figure S1: Average Rarefaction Plots Summarized for Sample Type. Rarefaction plots were constructed by subsampling 175 sequences from the rarefied OTU table ten times and plotting the number of observed OTUs after each subsampling.

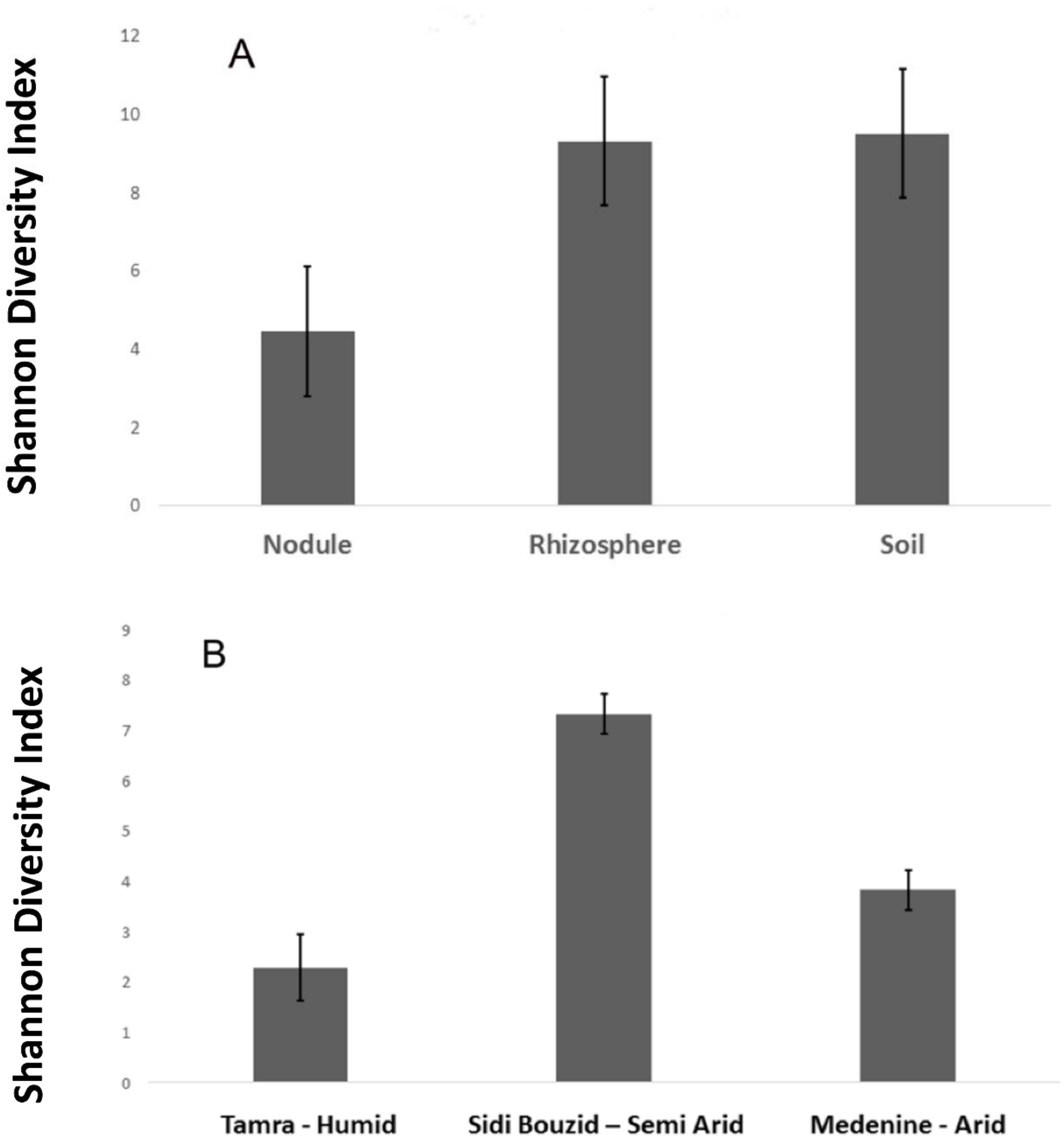

Figure S2: Alpha Diversity using the Shannon Diversity Index. (A) Alpha diversity of sample types. Asterisks demark that the nodule samples were significantly less diverse than both the rhizosphere and soil samples (One-tailed T-test P < 0.5). (B) Alpha diversity of nodules by site. All nodule sample sites differed significantly from another using the one-tailed Students T-Test (P < 0.05)

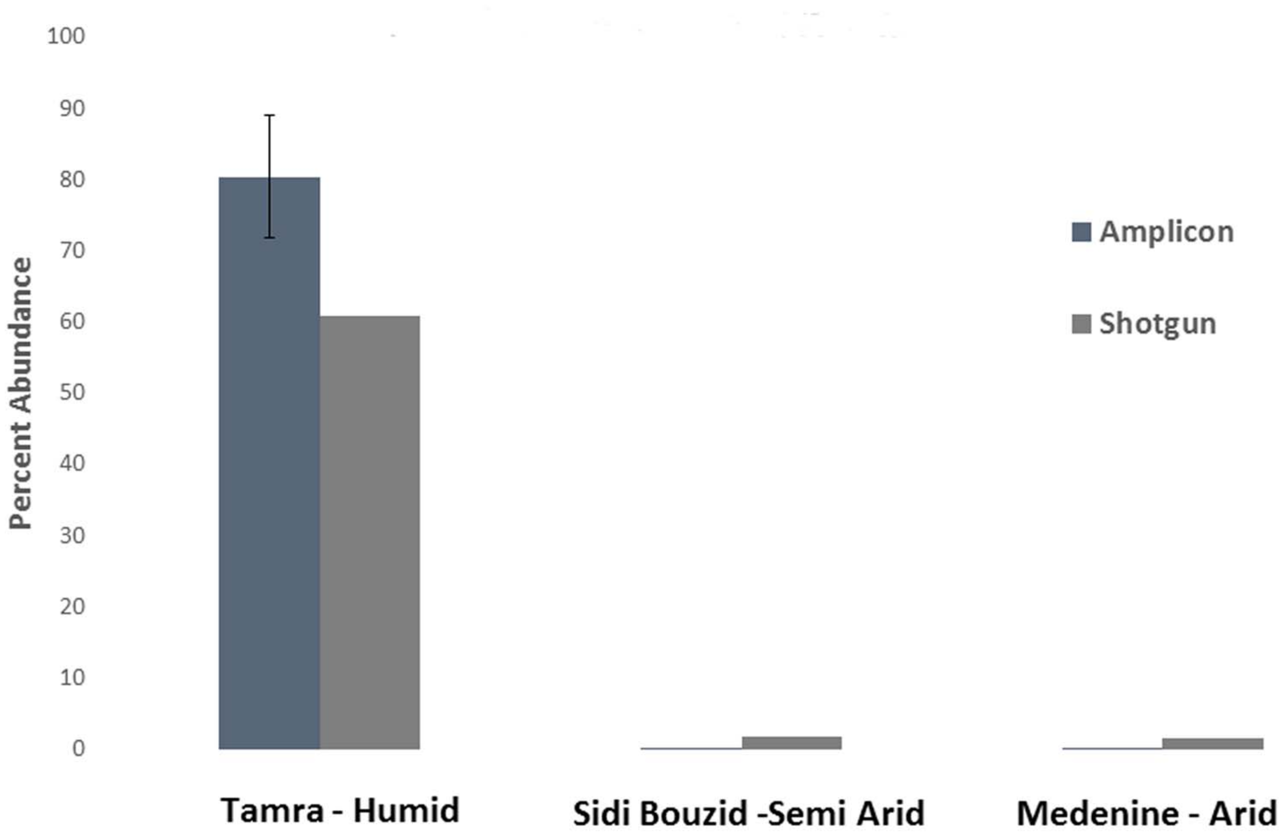

Figure S3: Relative Abundance of *Frankia* in Root Nodule Samples. Amplicon data refers to average relative abundance of *Frankia* after 16s copy number correction. Shotgun data refers to the percentage of read sets that aligned to the binned *Frankia* genome.

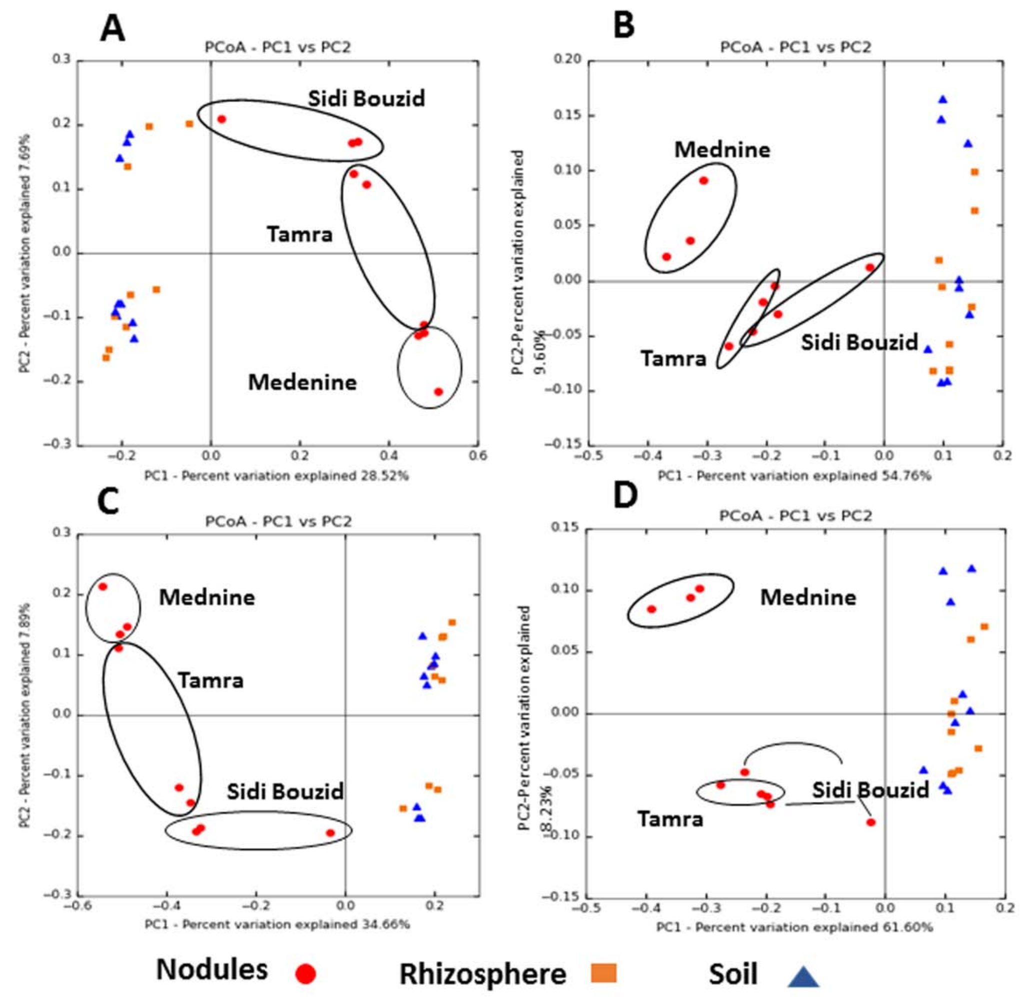

Figure S4: Principal Coordinates Ordination of CSS Normalized UniFrac Distance Matrices: Ordinations **A** and **B** where normalized using Cumulative Sum Scaling. Ordinations **C** and **D** were first adjusted for 16s copy number variation and then normalized by Cumulative Sum Scaling. Ordinations **A** and **C** were made using the unweighted UniFrac distance and ordinations **B** and **D** were made using the weighted UniFrac distance. For all ordinations MRPP showed a statistically significant grouping of the nodule samples, as outlined in Table S4.

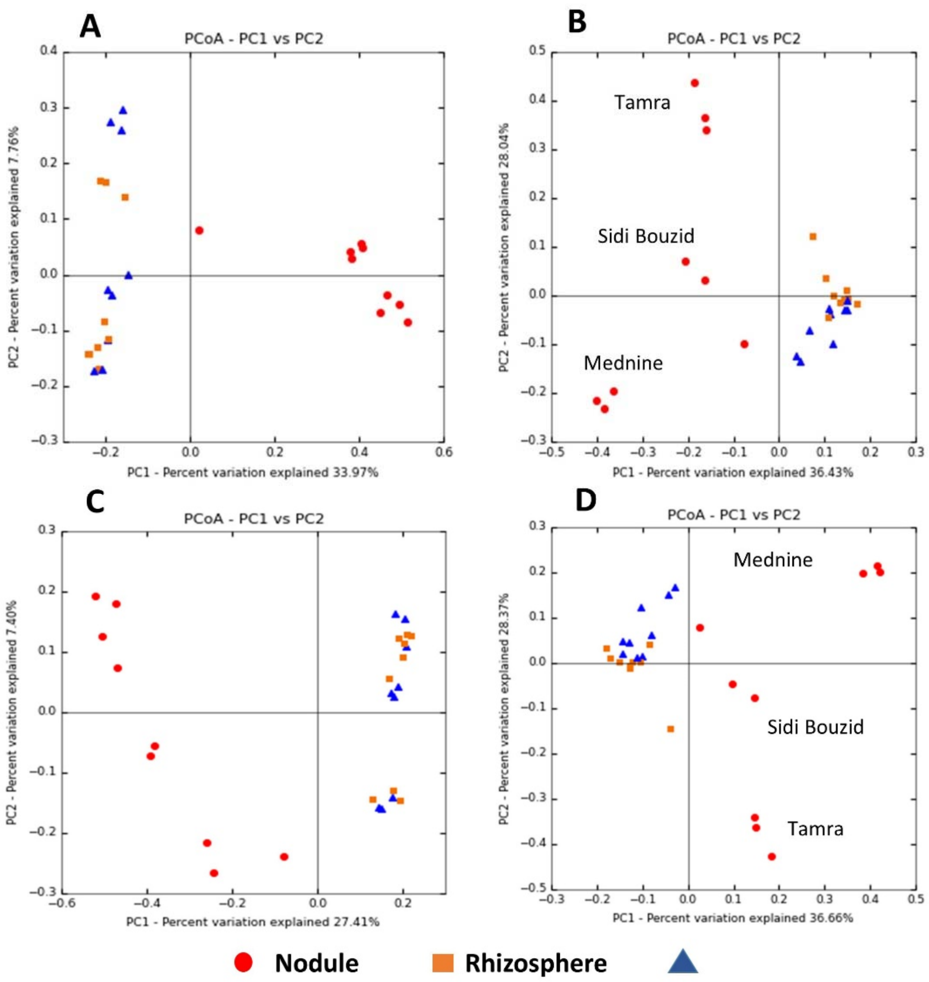

Figure S5: Principal Coordinates Ordination of UniFrac Distance Matrices: Ordinations **A** and **B** were produced from raw OTU tables that were 16s copy number corrected. Ordinations **C** and **D** were produced from evenly rarefied OTU tables. Ordinations **A** and **C** were made using the unweighted UniFrac distance and ordinations **B** and **D** were made using the weighted UniFrac distance. For all ordinations MRPP showed a statistically significant grouping of the nodule samples, as outlined in Table S4. Geographical sampling site labels on B and D are there to show that the nodules to separate along the second PcoA axis, which explains the second most amount of taxonomic variation in the data set.

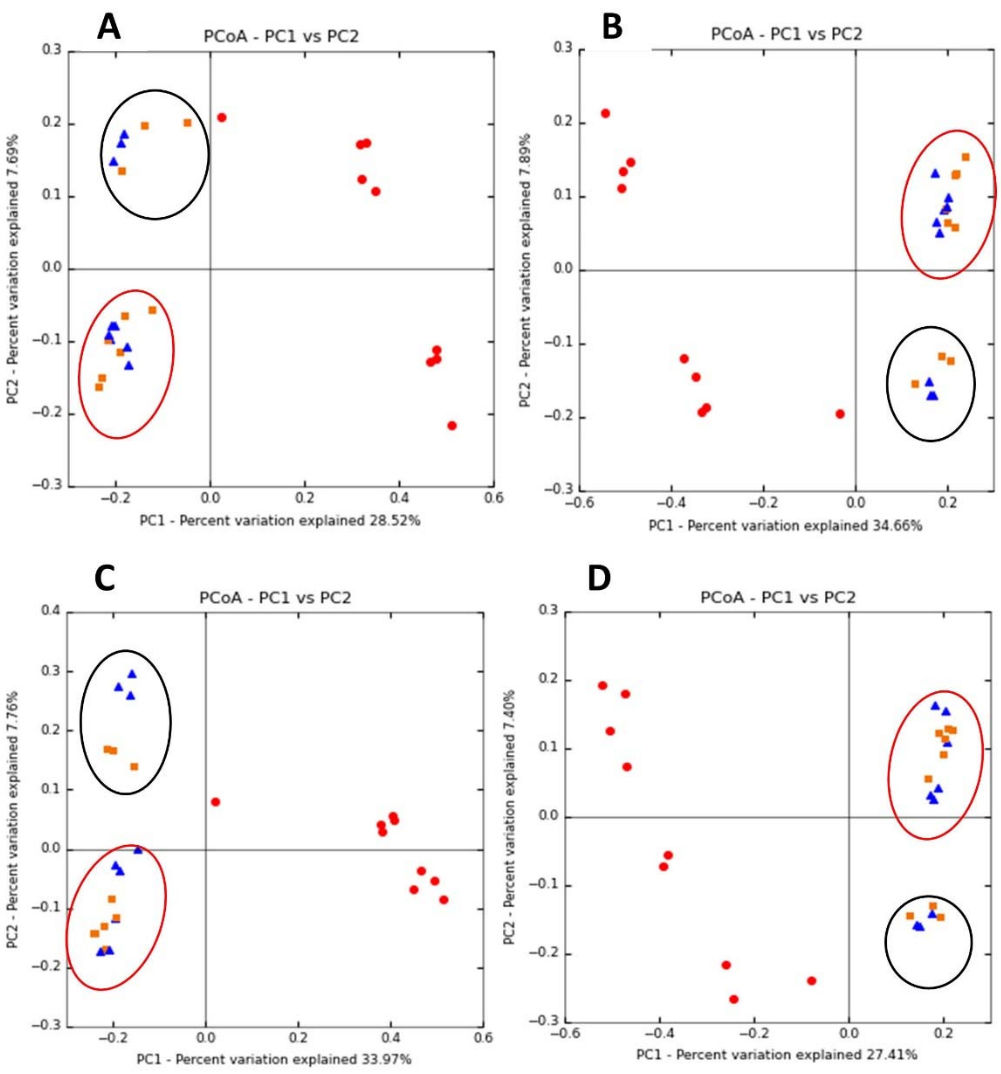

Figure S6: Principal Coordinate Ordinations of Unweighted UniFrac Distance Matrices. For all ordinations, samples grouped by black circles are soil and rhizosphere samples from the humid sampling site and samples grouped by red circles are samples from the semi-arid and arid sampling sites. **A:** Ordination from distance matrix produced from OTU table that was normalized by Cumulative Sum Scaling. **B:** Ordination from distance matrix that was produced from OTU table that was corrected for 16s operon copy number before Cumulative Sum Scaling. **C:** Ordination from raw OTU table that was corrected for 16s operon copy number. **D.** Ordination from distance matrix that was produced from evenly rarefied OTU table. MRPP statistics for these ordinations can be seen in Table S6.

Table S1. Enzyme Commission (EC) Numbers used for mining metagenome for potential Indole Acetic Acid Biosynthetic genes.

**Pathway**	**Enzyme**	**Enzyme Commission Number(s)**
IPyA	Tryptophan-pyruvate aminotransferase	2.6.1.99, 2.6.1.27
IPyA	Indole-3-Pyruvate decarboxylase	4.1.1.74
IPyA	Indole-3-acetaldyde dehydrogenase	1.2.1.3
IAM	Tryptophan monooxygenase	1.13.12.3
IAM	Indole-3-acetamide hydrolase	3.5.1
TAM	Tryptophan decarboxylase	4.1.1.28
TAM	Amine oxidase	1.4.3.21
IAN	Arylacetonitrilase	3.5.5.5
PAA	Phenylalanine aminotransferase	2.6.1.58
PAA	Phenylpyruvate decarboxylase	4.1.1.43
PAA	Phenylacetaldehyde dehydrogenase	1.2.1.39

Table S2. Enzyme Commission (EC) numbers used for mining Chitin, Pectin and Cellulose degrading genes.

**Enzyme**	**Mechanistic Action**	**Enzyme Commission Number(s)**
Chitinase	Cleavage of Glycosidic Bonds	3.2.1.14
Pectinase	Cleavage of polysaccharide bonds in pectin	3.3.1.15, 4.2.2.2
Exocellulase	Cleavage of small polysaccharides off the ends of polymer chains	3.2.1.91
Endocellulase	Cleavage of internal bonds in cellulose polymers	3.2.1.4
Beta-glucosidase	Hydrolyze product of exocellulases into monosaccharides	3.2.1.21
1,4-beta-glucosidase	Remove of glucose units from glucans and other oligosaccharides	3.2.1.74
Cellobiohydrolase	Hydrolysis of (1-4)-beta-D-glucosidic linkages in cellulose	3.2.1.176

Table S3. Enzyme Commission (EC) numbers used for mining Alkaline Phosphatase, Phytase and ACC Deaminase genes.

**Enzyme**	**Action**	**Enzyme Commission Number**
Alkaline Phosphatase	Dephosphorylation, Phosphate solubilization	3.1.3.1
3-Phytase	Cleavage of phytic acid	3.1.3.8
4-Phytase	Cleavage of phytic acid	3.1.3.26
5-Phytase	Cleavage of phytic acid	3.1.3.72
1-Aminocyclopropane-1-Carboxylate Deaminase	Regulation of plant ethylene levels	3.5.99.7

Table S4. **Significance of Nodule Grouping by MRPP.** Statistics for Cumulative Sum Scaling (CSS) normalized, Rarefied and Relative Abundance with 16s copy number correction, using the Weighted Unifrac (WU) and Unweighted UniFrac (UW).

**Normalization Type**	**CSS - WU**	**CSS -UW**	**CSS-16s-WU**	**CSS-16s-UW**	**Rarefield-WU**	**Rarefield-UW**	**16s-Rel-WU**	**16s-Rel-UW**
A Statistic	0.2529	0.1138	0.2926	0.1451	0.177	0.1079	0.1832	0.1437
P Value	0.001	0.001	0.001	0.001	0.001	0.001	0.001	0.001

Table S5. **MRPP Results of pooled Soil and Rhizosphere samples**

**Normalization Type**	**Raw-Relative**	**16s Copy Number Relative**	**Rarefied**	**CSS**	**16s Copy Number CSS**
A statistic	0.03552	0.03858	0.0323	0.01597	0.0233
P Value	0.063	0.035	0.049	0.167	0.104

Table S6. **MRPP results for Humid-Arid grouping in ordinations seen in Figure S2.**

**Normalization Type**	**Raw-Relative**	**16s Copy Number Relative**	**Rarefied**	**CSS**	**16s Copy Number CSS**
A Statistic	0.06809	0.07799	0.05062	0.06019	0.07526
P Value	0.001	0.001	0.001	0.001	0.001

Table S7. **Assembly Statistics from Metagenome Assembly**

Feature	Number
Number of contigs ≥ 1,000 bp	122,849
Total length of contigs ≥ 1,000 bp	365,121,128
Number of contigs ≥ 500 bp	432,339
Total length of contigs ≥ 500 bp	569,464,861
Largest contig	1,267,956
GC (%)	47.20
N_{50}	1,683
